# A Chinese Traditional Therapy for Bleomycin-Induced Pulmonary Fibrosis in Mice

**DOI:** 10.1155/2018/8491487

**Published:** 2018-09-18

**Authors:** Lifang Sun, Minjie Mao, Zhisheng Yan, Cuiyun Zuo, Xiaojie Zhang

**Affiliations:** ^1^Department of Tuberculosis, Hangzhou Red Cross Hospital, Hangzhou 310003, China; ^2^Department of Critical Care Medicine, Pingdu People's Hospital, Qingdao 266700, China; ^3^Department of Respiratory Disease, Third Hospital of Xiamen, Xiamen 361100, Fujian, China; ^4^Department of Emergency Medicine, Hangzhou Red Cross Hospital, Hangzhou 310003, China

## Abstract

Pulmonary fibrosis is a chronic and fatal disease of lung tissue with high incidence and mortality in the world. The exploration of effective treatment for pulmonary fibrosis remains an urgent challenge. In our study, Qingfei Xieding was investigated as a novel Chinese traditional patent medicine against pulmonary fibrosis. A pulmonary fibrosis mouse model was constructed by injecting with bleomycin sulfate. Following Qingfei Xieding administration, lung samples were collected to assess pulmonary phenotype changes by analyzing lung coefficient, wet/dry, and histopathologic section. Levels of nitric oxide (NO), hydroxyproline (HYP), malondialdehyde (MDA), and total antioxidant capacity were measured to evaluate the degree of oxidation. A single-cell gel electrophoresis (SCGE) assay was used to evaluate bleomycin-induced DNA damage. Western blotting and real-time quantitative PCR were performed to determine the abundance of inducible nitric oxide synthase (iNOS), connective tissue growth factor (CTGF), alpha smooth muscle actin (α-SMA), and fibronectin (FN). In the present study, Qingfei Xieding administration significantly attenuated bleomycin-induced pulmonary fibrosis in mice by reducing lung coefficient, wet/dry, NO, HYP, and MDA as well as the expression of iNOS, CTGF, *α*-SMA, FN, and DNA damage. The results indicated that Qingfei Xieding is effective to resist oxidative damage and histopathologic lesion, serving a protection role on bleomycin-induced pulmonary fibrosis.

## 1. Introduction

Pulmonary fibrosis is a chronic lung disease that is characterized by chronic inflammation destruction, fibroblast proliferation, lung tissue damage, and extracellular matrix deposition [1, 2]. With a series of severe respiratory impairments, its incidence and mortality have increased in the past ten years all over the world [3]. More than one half of the patients of pulmonary fibrosis have idiopathic pulmonary fibrosis (IPF), which is a fatal disease with low survival rate [3, 4]. The current and popular therapeutic approaches involve treatments with corticosteroids, antifibrotic drugs, immune-suppressant, and cytotoxin [5, 6], but clinical research studies have shown that those therapies are of limited efficacy and often incur intolerable side effects. Therefore, the exploration of treatment strategies plays a great role on the successful cure.

Chinese traditional patent medicines (CTPMs) were viewed as the essence of Chinese medicine from ancient medical scientist after thousands of years of medical practice and creation, which were extracted from raw Chinese herbs and then manufactured into several forms, such as dripping pills, liquids, syrups, powders, granules, instant teas, cream, and capsules [7, 8]. With large numbers of clinical and experimental research studies, many Chinese classical formulas are made into patent medicines and approved for their efficacy, safety, and convenience. It is wildly applied as complementary or adjunctive therapies for symptom management and body maintaining. Therefore, more and more CTPMs for specific diseases are approved and used clinically, such as Liuwei Dihuang Wan, a popular honey pill for nourishing yin and kidney and strengthening the body [9]. Recent studies showed that Shenmai injection, Wenxin granule, and Shensongyangxin capsule can prevent ischemic stroke in patients with atrial fibrillation [10]. Zilongjin tablet, Liqi, as antitumor drugs, improves quality of life in cancer patients [11].

Qingfei Xieding prescription, a novel self-developed CTPM of our hospital, is a concentrated granule purified from herbs, including Ephedra sinica, apricot kernel, gesso, lobed kudzuvine root, Scutellaria baicalensis, Bombyx batryticatus, and Houttuynia cordate . The main ingredients of Qingfei Xieding are Houttuynia cordate and Scutellaria baicalensis, which exhibit multiple beneficial properties, such as antiviral, anti-inflammatory, antibacterial, antioxidant, antitussive, and immune-strengthening activities [12–14]. It has been used to overcome clinical pulmonary tuberculosis and achieve good therapeutic effect. According to our clinical case investigation, a combination treatment of Qingfei Xieding prescription and Western therapy has helped about 10 thousands of patients suffering from lung diseases every year, while the mechanism of its function is still poorly understood. Therefore, these backgrounds encourage us to further elucidate the potential role of Qingfei Xieding on pulmonary injury. Here, we established the mouse model of pulmonary fibrosis via exposure to bleomycin, which was a most effective antineoplastic chemotherapeutic agent. However, bleomycin was often used as disease inducer of pulmonary fibrosis due to its serious side effect on lung [15]. Bleomycin-induced pulmonary fibrosis had been regarded as one of the widely used fibrosis models because of pathologic changes caused by bleomycin those were similar to lung fibrosis of human being [16–18]. In the present research, we aimed at investigating the effect of Qingfei Xieding on the development of pathological process and its molecular regulation by utilizing the bleomycin-induced pulmonary fibrosis model.

## 2. Materials and Methods

### 2.1. Animals, Chemicals, and Reagents

Mature C57BL/6 mice with 8-week-old was supplied by Guangdong Medical Laboratory Animal Center (license number: SYXK(Yue)2008–0002) and maintained in a standard pathogen-free environment with a controlled diet and a controlled temperature (23–25)°C. Laboratory animal experiments performed were approved by the Hangzhou Red Cross Hospital Animal Care and Use Committee.

Qingfei Xieding was available from Hangzhou Red Cross Hospital. The mixture of Ephedra sinica (9 g), apricot kernel (9 g), gesso (18 g), lobed kudzuvine root (9 g), Scutellaria baicalensis (9 g), Bombyx batryticatus (15 g), and Houttuynia cordate (3 g) was decocted in 1.5 L water till condensing to 300 ml. After removing the dregs, extracts were collected and stored at 4°C.

Bleomycin sulfate was purchased from Sigma (St. Louis, MO, USA). HYP, NO, T-AOC, and MDA assay kits were obtained from Beyotime (Jiangsu, China). Antibodies used in this study were purchased from Abcam (Cambridge, British). RNeasy mini kit and qRT-PCR assay kit were obtained from Qiagen (Shanghai, China) and Takara (Dalian, China), respectively.

### 2.2. Pulmonary Fibrosis Mouse Model and Qingfei Xieding Administration

Forty 8-week-old female mice, weighing 20–25 g, were randomly divided into the following four groups: saline-water; BLM-water; BLM-Qingfei; saline-Qingfei. The BLM group mice were intratracheally injected with bleomycin sulfate (5 mg/kg mouse in 50 *μ*l saline) for 7 days to induce mice with pulmonary fibrosis. The therapeutic effect of Qingfei Xieding on bleomycin-induced pulmonary fibrosis was evaluated by gavaging Qingfei Xieding (12 ml/kg) for 28 days.

### 2.3. Lung Coefficient Calculation

Twenty-eight days after Qingfei Xieding treatment, mice were anesthetized with 10% chloral hydrate (350 mg/kg) and sacrificed. Mouse lungs were removed and weighed as body weight. The lung surface was flushed with saline, and the blood was removed, weighed, and recorded as lung wet weight. Lung coefficient = lung wet weight (mg)/body weight (g). The lung dry weight was measured after incubating the samples at 100°C for 48 h.

### 2.4. Histopathologic Evaluation

After evaluating the lung coefficient, the left lungs of the mice were used to evaluate the fibrotic score by histological examination, and the right lungs were then homogenized to analyze the expression of iNOS, CTGF, *α*-SMA, and FN. For histopathologic evaluation, the left lung of each mouse was fixed in 4% paraformaldehyde (Dissolved in PBS) and processed for routine paraffin embedding. After embedding in paraffin, the tissue sections (5 *μ*m) were cut and stained with hematoxylin and eosin (HE) and examined by light microscopy, a modified Masson trichrome to assess the degree of fibrosis.

### 2.5. Hydroxyproline (HYP) and Total Antioxidant Capacity (T-AOC) Detection

Mice lung tissue samples were homogenized in cold PBS at 4°C with a Polytron homogenizer. HYP and T-AOC assay kits were used to detect the HYP and T-AOC levels in lung tissue homogenate samples.

### 2.6. Malondialdehyde (MDA) and Nitric Oxide (NO) Detection

Twenty-eight days after the initial injection of bleomycin, mice were anesthetized with 10% chloral hydrate. Mice abdomens were open with median incision, and blood plasma was collected by the abdominal aortic method using a disposable syringe. The plasma was prepared and stored at −80°C until assayed. MDA and NO assay kits were used to measure the levels in plasma.

### 2.7. RNA Isolation and Real-Time Quantitative PCR (qRT-PCR)

Total RNA of fresh lung tissues (40 mg per sample) were extracted as Qiagen RNeasy mini kit described. According to the Takara qRT-PCR assay kit instruction, the cDNA was produced with the total RNA, and PCR reaction (primers listed in Table 1) was performed as follows: one cycle of 94°C for 5 min; then 40 cycles of 94°C for 15 s, 58°C for 30 s, and 72°C for 20 s; and one cycle of 72°C for 10 min. Relative abundance of genetic transcription was obtained by calculating the comparative threshold cycle.

### 2.8. Western Blot Analysis of iNOS, CTGF, α-SMA, and FN Expression

Mice lung tissue samples were homogenized in cold lysis buffer (50 mM Tris-HCl, pH 7.4, 5 mM EDTA, 0.5%NP-40, 150 mM NaCl) supplemented with 1x protease/phosphatase inhibitor cocktail (Cell Signaling Technology). Equal protein amounts of tissue lysates were loaded. Proteins were separated by SDS-PAGE gel and transferred to PVDF membranes. After blocking in 5% milk in TBST (10 mM Tris-HCl (pH 7.4), 150 mM NaCl, and 0.1% (v/v) Tween-20) for 1 h, the membranes were incubated with iNOS, CTGF, *α*-SMA, and FN antibodies, respectively, overnight at 4°C, followed by the second antibody. The protein expression was determined using Lumigen ECL Ultra Western Blotting HRP Substrate (Lumigen), and the signals were detected by Fujifilm Luminescent Image Analyzer (LAS4000).

### 2.9. Single-Cell Gel Electrophoresis (SCGE)

The SCGE assay was performed as described by Tice & Vasquez with some modifications. The remaining 20* μ*L of cells was placed in 150 *μ*L of low melting point agarose (0.5%; Sigma, St. Louis, MO) and spread onto two microscope slides that had been prelayered with 180 *μ*L of normal melting point agarose (1%; Sigma, St. Louis, MO); the slides were then immersed in a chilled lysis solution (2.5 M·NaCl, 100 mM Na_2_EDTA, 10 mM Tris at pH 10, 10% DMSO, 1% Triton X-100 fresh; Sigma, St. Louis, MO). After lysis at 4 °C for 1 h, the slides were placed on a horizontal electrophoresis unit (Gibco BRL Life Technologies Inc.). The DNA was allowed to unwind for 20 min in electrophoresis running buffer solution (300 mM·NaOH, 1 mM·Na_2_EDTA at pH >13; Sigma, St. Louis, MO). Electrophoresis was conducted for 20 min at 25 V and 300  mA (0.73 V/cm). All the technical steps were carried out under very dim indirect lighting. After electrophoresis, the slides were gently removed and rinsed with neutralization buffer (0.4 M·Tris pH 7.5, Sigma, St. Louis, MO); they were then dehydrated with ethanol (70%) and air-dried. All of the slides were coded before analysis. Ethidium bromide (75 *μ*L of 20 *μ*g/mL solution) was added to each slide, and a cover glass was placed on the gel. Individual nuclei were visualized under 400x magnification on a Leica DM4000 microscope.

### 2.10. Statistical Analysis

The data were analyzed by one-way analysis of variance (ANOVA) with a Tukey's post hoc test using GraphPad Prism 5. The data were expressed as the mean value ±standard deviation (SD). Significance was considered at *P* < 0.05.

## 3. Results

### 3.1. Lung Coefficient, Wet/Dry, and Hydroxyproline Level

To assess the state of lung in each group, lung coefficient and wet/dry (W/D) of lungs were detected after the treatment of Qingfei Xieding. As shown in Table 2, BLM-treated model groups displayed higher lung coefficient and W/D than those of control groups, but control Qingfei groups had no significant change, similar to control groups. Treatment with Qingfei Xieding reduced the bleomycin-induced increase in lung coefficient and wet/dry, while it was unable to restore normal level, especially lung coefficient.

Determination of hydroxyproline concentration could provide useful information for the diagnosis and prognosis of pulmonary fibrosis caused by disorders of collagen metabolism, since hydroxyproline is a significant biomarker of collagen deposition. The content of hydroxyproline in the BLM-treated model lungs raised approximately two-fold compared with that in the control groups. Qingfei Xieding given to model groups contributed to the significant decrease of lung collagen content triggered by bleomycin, but it has no adverse influence on that of control groups.

### 3.2. Malondialdehyde, Nitric Oxide, and Total Antioxidant Capacity

To elucidate the relationship between bleomycin-induced oxidative damage and function of Qingfei Xieding, oxidation stress level and antioxidation capacity in lungs of each groups were estimated by the levels of malondialdehyde, nitric oxide, and the total antioxidant (Table 3). Bleomycin led to about three-fold increase in malondialdehyde and nitric oxide compared with those in the control groups or control Qingfei groups. Qingfei Xieding decreased the extent of bleomycin-induced oxidative stress, eliminated oxidants, and enhanced antioxidation, which was corresponding to the result of total antioxidant activity as shown.

### 3.3. Histopathological Examination

**Figure 1:**
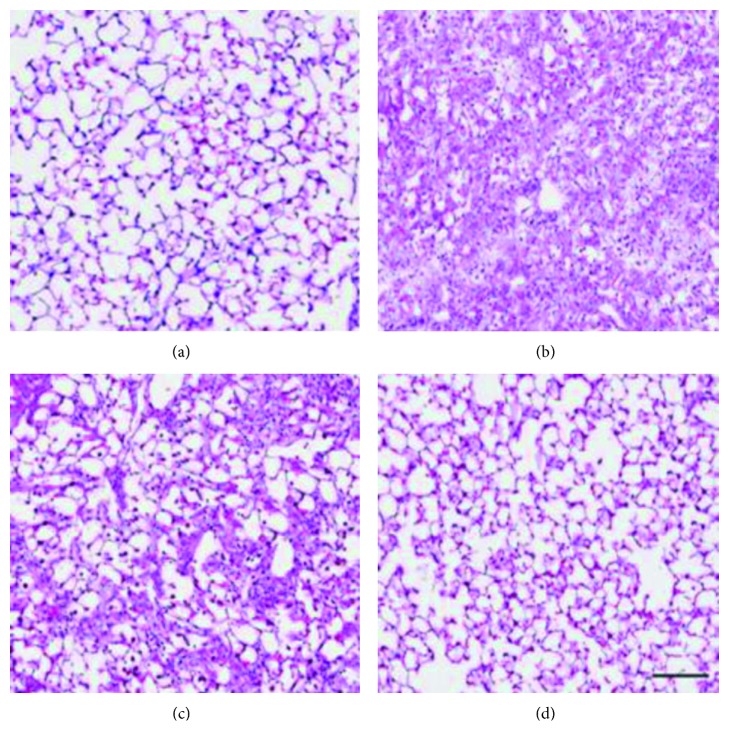
Effect of Qingfei Xieding on histopathologic changes of lung-induced by bleomycin in mice. (a) Saline-water-treated rat; (b) BLM-water-treated rat; (c) BLM-Qingfei-treated rat; (d) saline-Qingfei-treated rat. Compared with the animals of the BLM-water group, lung histologic findings on the BLM-Qingfei group showed less fibrotic lesions. All panels were stained with HE and are shown at the same magnification. Scale bar, 100 *μ*m.

To further evaluate changes to pulmonary phenotype, lung tissue sections from the four groups were stained with hematoxylin and eosin. As shown in Figure 1, sections from the control groups exhibited normal structure without any pathologic changes. The intratracheal injection of bleomycin led to the extensive proliferation of fibroblasts, the infiltration of inflammatory cells, the prominent deposition of fibrillar collagen, and the destruction of normal pulmonary architecture. Impressively, the administration of Qingfei Xieding could significantly reduce these pathological lung damages, such as fibrosis and inflammation.

### 3.4. Expression of iNOS, CTGF, α-SMA, and FN

**Figure 2:**
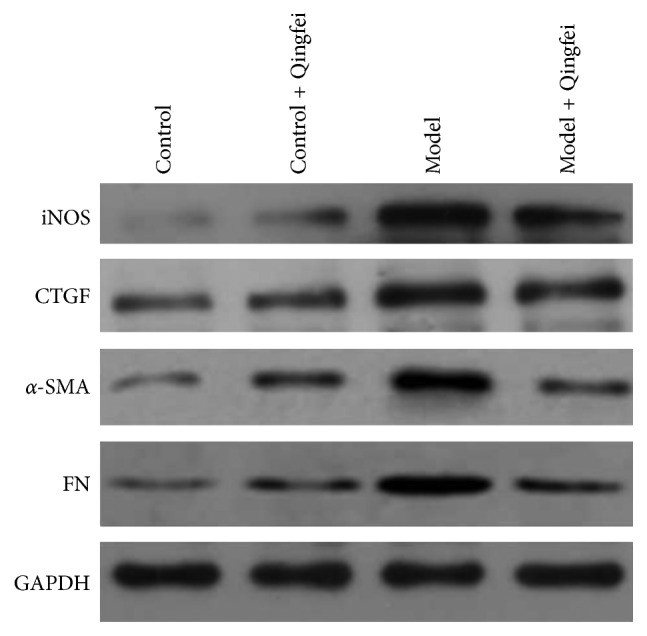
Western blotting analysis for iNOS, CTGF, *α*-SMA, and FN expression in lung tissues. GAPDH, a housekeeping gene, was selected as the internal reference to determine the same sample loading.

To identify the correlation between the related signaling pathway and effect of Qingfei Xieding on pulmonary fibrosis, the free radical nitric oxide synthase and the pathological markers of collagen deposition were examined by western blotting. As shown in Figure 2, compared with the normal control group given water, bleomycin resulted in the elevation of the expression level for iNOS, CTGF, *α*-SMA, and FN. However, Qingfei Xieding attenuated the bleomycin-induced increase, and there were no statistical differences between model Qingfei group, control Qingfei group, and control group.

### 3.5. DNA Damage

**Figure 3:**
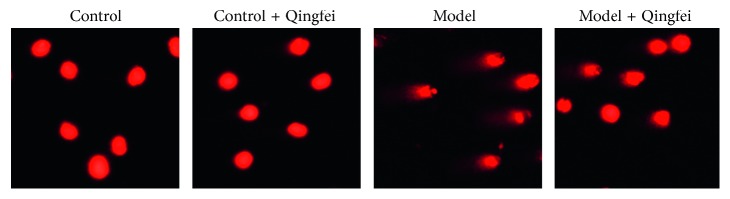
Single-cell gel electrophoresis (SCGE) observed the DNA damage.

SCGE assay was performed to measure DNA strand breaks at the level of individual cells from different lung samples. The evidence in Figure 3 showed that bleomycin resulted in DNA broken strands and forming comet tails, which was reflected by the fluorescence intensity of DNA in the head and tail. However, administration of Qingfei Xieding protected the cells from DNA damage by bleomycin quantified as diminishing comet tail length.

## 4. Discussion

Bleomycin, discovered by Hamao Umezawain 1962, is a popular medication for chemotherapy that is mostly used in the treatment of squamous cell cancers, melanoma, sarcoma, testicular and ovarian cancer, Hodgkin's, and non-Hodgkin's lymphoma [15, 19]. It is thought to inhibit DNA synthesis by breaking its strands and also the synthesis of RNA and protein to a lesser degree, thereby preventing cell division and causing the tumor to shrink [20, 21]. The binding of bleomycin to DNA and iron induces production of ROS and initiates inflammatory changes through action of cytokines that lead to collagen accumulation in the lung [22, 23]. Therefore, the most serious complication of bleomycin in patients is pulmonary fibrosis and impaired lung, which is dependent on its usage and duration. So, bleomycin is wildly employed to build the animal models of pulmonary fibrosis and lung injury for preclinical medicine research studies [19, 24]. Our pathological study showed that 4-week after instillation of bleomycin into mice caused classic evidences of pulmonary fibrosis, but Qingfei Xieding alleviated the degree of fibroblast proliferation and subsequent collagen content and improved the development of lung pathological fibrosis. These data suggest that Qingfei Xieding exerts antifibrotic effects that may be useful in the treatment of pulmonary fibrosis.

Pulmonary fibrosis is a progressive pathological change of lung tissue, including the excessive deposition of extracellular matrix, the formation of chronic interstitial inflammation, proliferation of fibroblasts, and alveolar collapse [25]. HYP as the main component of collagen, accounting for about 13% of the total amino acids, is used as an important targeted indicator of collagen metabolism [25, 26]. During the early stage development of collagen abnormity, the abundance of HYP and the level of lipid peroxidation hallmarked by MDA would be on the rise [26, 27]. With further progression of pulmonary fibrosis, the expression of inducible nitric oxide synthase (iNOS) can accelerate the synthesis of endogenous free radical nitric oxide [28, 29], which can affect proteins, lipids, carbohydrates, and nucleic acids that are the main components of cells related to apoptosis, gene expression, signal transduction, and defense against pathogens. Growing number of papers on the different aspects of oxidant/antioxidant imbalance in lung diseases in the last decade reported that the pulmonary redox imbalance or the oxidative burden of nitric oxide was closely involved in lung tissue damage in IPF patients, and the overproduction of free radical reactions plays a key role in bleomycin-induced pulmonary inflammation and fibrosis [30, 31]. These studies provided important insights into pathogenesis and clue for the discovery of new treatments. Our study showed that bleomycin enhanced the concentrations of NO, HYP, and MDA in model mice, and Qingfei Xieding could resist its negative effects, suggesting that Qingfei Xieding may act as an antioxidant to protect the lung from the damage of bleomycin-induced oxidant.

**Figure 4:**
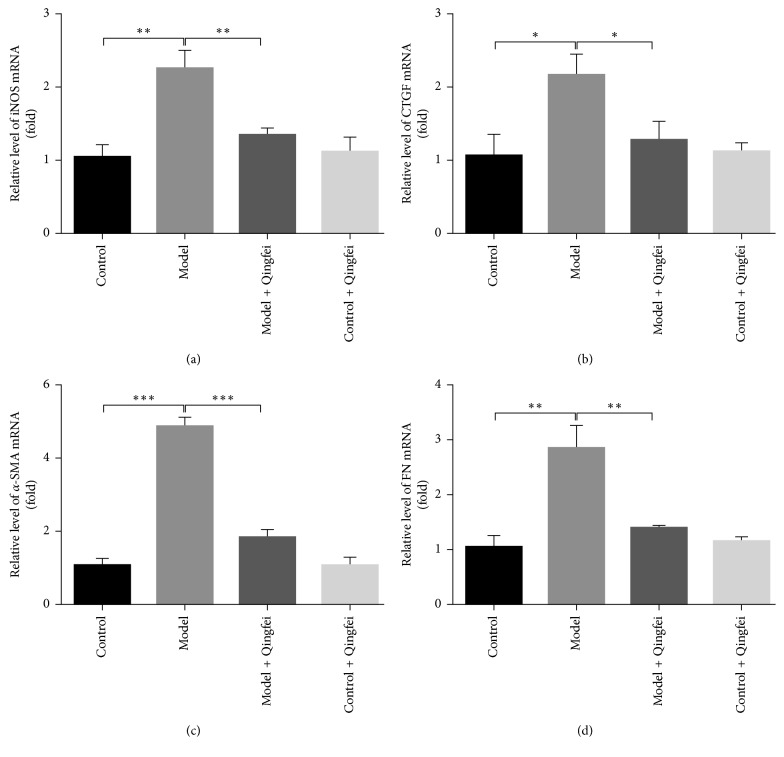
Relative transcriptional levels of iNOS, CTGF, *α*-SMA, and FN genes detected by qRT-PCR. Compared with the control group, the relative levels of the related genes were higher than those of the BLM model group. Qingfei Xieding down regulated the transcription of those pathway genes. ^*∗*^*P* < 0.05, ^*∗∗*^*P* < 0.01, and ^*∗∗∗*^*P* < 0.001 (versus the model).

Connective tissue growth factor (CTGF), a cysteine-rich extracellular matrix-associated secreted protein, has been proven to exist in numerous normal tissues at low levels and become significantly upregulated in the fibrogenetic and cancerous processes [28, 32]. Therefore, CTGF is regarded as a pathological marker of collagen deposition and pulmonary fibrosis. It acts as a downstream mediator or regulator of transforming growth factor beta (TGF-*β*) [32], which further promotes myofibroblast and stimulates the production of alpha smooth muscle actin (*α*-SMA) and fibronectin (FN) [33, 34]. A growing number of evidences demonstrated that the expression of CTGF, TGF-*β*1, and FN was synergistically activated and elevated in the lung tissues of patients and animal models with lung fibroblasts [35], suggesting that TGF-*β*/CTGF signal pathway may be a potential therapeutic target in treatments. Our present data revealed Qingfei Xieding can alter the expression levels of CTGF, TGF-*β*1, and FN induced by bleomycin and cause a protective response through regulating TGF-*β*1 signaling in a negative pattern among the model group, implying that Qingfei Xieding may play a crucial role in the cell proliferation and TGF-*β*1-stimulated genetic transcription, which was consistent with our relative transcriptional data in Figure 4.

## 5. Conclusions

In summary, our observations demonstrated that Qingfei Xieding, as a Chinese traditional patent medicine, has protective effects against pulmonary fibrosis induced by bleomycin. We further confirmed that Qingfei Xieding is effective to scavenge oxidative damage of the lung caused by reactive oxygen species and inhibit TGF-*β*/CTGF-stimulated pathway. Therefore, the present study implies that Qingfei Xieding may facilitate the treatment of bleomycin-induced pulmonary fibrosis and serve as a potential medicament for lung disease.

## Figures and Tables

**Table 1:** Primers used in this study.

Gene	Accession number	Sequences
CTGF	NM_010217	Forward: 5'AGCCAACTGCCTGGTCCAGA3'
		Reverse: 5'CGCAGAACTTAGCCCTGTATGT3'
*α*-SMA	BC064800	Forward: 5'TCCTTCGTGACTACTGCCGAGC3'
		Reverse: 5'AATGGTGATCACCTGCCCGTC3'
iNOS	BC062378	Forward: 5'ACTTCCAATGCAACATGGGAGC3'
		Reverse: 5'GTGGTGCGGCTGGACTTTTC3'
FN	BC145271	Forward: 5'TCTGGATACCGTGTGGAGGTCC3'
		Reverse: 'CTGACAGGCAGGACCTCCACA3'
GAPDH	NM_008084	Forward: 5'TGTGGATGGCCCCTCTGGAA3'
		Reverse: 'TTGGCAGGTTTCTCCAGGCG3'

**Table 2:** Effect of Qingfei Xieding on lung coefficient, W/D, and HYP following bleomycin-induced lung fibrosis in rats.

Group	Lung coefficient (g/kg)	W/D (g/g)	HYP (*μ*g/g)
Control	6.89 ± 0.23	3.81 ± 0.56	269.23 ± 2.11
Model	18.26 ± 0.88^*∗∗∗*^	5.63 ± 0.48^*∗∗*^	512.32 ± 3.15^*∗∗∗*^
Model + Qingfei	10.35 ± 0.65^*∗∗*^^##^	4.19 ± 0.22^*∗*^^#^	300.23 ± 4.15^*∗∗*^^###^
Control + Qingfei	6.64 ± 0.68	3.75 ± 0.33	268.96 ± 1.32

Versus normal: ^*∗*^*P* < 0.05,^*∗∗*^*P* < 0.01, and ^*∗∗∗*^*P* < 0.001; versus the model: ^#^*P* < 0.05, ^##^*P* < 0.01, and ^###^*P* < 0.001.

**Table 3:** Detection of NO, T-AOC, and MDA in lung tissues or serum of different rat groups.

Group	NO (*μ*mol/g)	T-AOC (k*μ*·g^−1^ protein)	MDA (*μ*mol/L)
Control	1.64 ± 0.54	0.93 ± 0.06	6.88 ± 0.47
Model	5.07 ± 0.63^*∗∗∗*^	0.45 ± 0.12^*∗∗*^	20.9 ± 0.99^*∗∗∗*^
Model+Qingfei	2.16 ± 0.38^*∗*^^##^	0.89 ± 0.25^*∗*^^#^	9.56 ± 0.85^*∗∗*^^###^
Control+Qingfei	1.72 ± 0.74	0.90 ± 0.09	6.79 ± 1.01

Versus normal: ^*∗*^*P* < 0.05, ^*∗∗*^*P* < 0.01, and ^*∗∗∗*^*P* < 0.001; versus the model: ^#^*P* < 0.05, ^##^*P* < 0.01, and ^###^*P* < 0.001.

## Data Availability

The data used to support the findings of this study are included within the article.
